# Branched RNA: A New Architecture for RNA Interference

**DOI:** 10.4061/2011/586935

**Published:** 2011-03-06

**Authors:** Anna Aviñó, Sandra M. Ocampo, José Carlos Perales, Ramon Eritja

**Affiliations:** ^1^Institute for Research in Biomedicine (IRB Barcelona), Institute of Advanced Chemistry of Catalonia (IQAC-CSIC), Networking Centre on Bioengineering, Biomaterials, and Nanomedicine (CIBER-BBN), Baldiri Reixac 10, 08028 Barcelona, Spain; ^2^Department of Physiological Sciences, School of Medicine, University of Barcelona (Campus Bellvitge), Feixa Llarga, L'Hospitalet de Llobregat, 08907 Barcelona, Spain

## Abstract

Branched RNAs with two and four strands were synthesized. These structures were used to obtain branched siRNA. The branched siRNA duplexes had similar inhibitory capacity as those of unmodified siRNA duplexes, as deduced from gene silencing experiments of the TNF-*α* protein. Branched RNAs are considered novel structures for siRNA technology, and they provide an innovative tool for specific gene inhibition. As the method described here is compatible with most RNA modifications described to date, these compounds may be further functionalized to obtain more potent siRNA derivatives and can be attached to suitable delivery systems.

## 1. Introduction

In recent years, siRNAs have generated tremendous interest in therapeutics [1]. Nevertheless, the transition of siRNAs from the laboratory to the clinical practice has encountered several obstacles. Briefly, siRNA duplexes are rapidly degraded in serum by exonucleases and endonucleases [2]. The polyanionic phosphodiester backbone of siRNA suffers from difficult cell uptake [3], and oligonucleotides may have off-target effects, either by stimulating the immune system [4] or by entering other endogenous gene regulation pathways [5]. Several chemical modifications have been proposed in the literature to address these drawbacks [2–4]. Most of these modifications are based on modified nucleosides and changes on backbone linkages [6, 7]. Thus, changes in sugar moiety influences sugar conformation, and, therefore, overall siRNA structure. Modifications of the 2′-OH by F or OMe as well as LNA [8, 9] are well tolerated and improve binding affinity and nuclease resistance. Base modifications that stabilize base pairs (5-bromouracil, 5-methylcytosine, 5-propynyluracil, and others) have also been proposed [7, 10]. Terminal conjugates, especially at the termini of the sense strand, have been modified with a large number of lipids to achieve improved cellular uptake [11].

**Figure 1:**
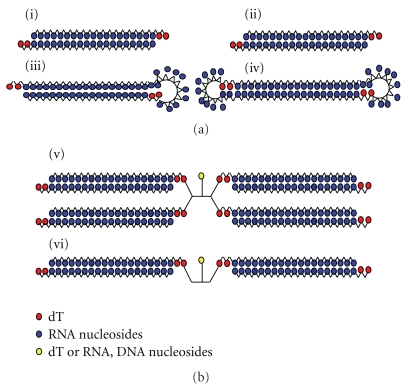
Duplex RNA architectures for RNA interference. (a) Described previously in the introduction: (i) canonical siRNA antiparallel duplex; (ii) small internally segmented interfering RNA (sisiRNA); (iii) small hairpin RNA (shRNA); (iv) dumbbell siRNA. (b) Branched siRNA described in this study: (v) four-stranded RNA; (vi) two-stranded RNA.

In addition to these modifications, siRNA architecture is also crucial in the design of effective and specific siRNA. The architecture itself can be altered by chemical synthesis. In addition to the canonical siRNA architecture of 21-nt antiparallel, double-strand RNA with 2-nt 3′-overhangs [12], several forms of siRNA have been described. Blunt-ended siRNA [13], 25/27 mer Dicer-substrate or asymmetric siRNA [14] are among the siRNA structures formed by two strands. Moreover, functional siRNA can also be formed by one single RNA strand. This is the case in small hairpin RNA (shRNA), where the two strands are linked by a single loop [15], or RNA dumbbells [16], made by closing the open end of the hairpin. This last structure retains RNAi activity while providing complete protection from nucleases [16]. Finally, siRNA can also comprise three strands, namely, two 9–13 nt sense strands and the intact antisense strand. This structure is known as small internally segmented interfering RNA [17] (sisiRNA). Some of these modifications have reduced off-target effects and increased potency (Figure 1(a)). Another architecture not yet explored in siRNA is the branched RNA structure obtained from a central building unit and several branching points that enable the strand growth.

Several strategies can be used to prepare branched RNA structures. Although the synthesis of these compounds is complex and tedious, commercially available synthons have improved the complexity and yields of these structures. 

The assembly of branched nucleic acids on a solid support can be achieved by convergent or divergent strategies. In the former, synthesis of branched oligonucleotides containing two or more identical strands can be achieved by branching derivatives 1,3-diaminopropanol, pentaerythritol, the commercially available symmetric doubler [18–20], or by a ribonucleoside bisphosphoramidite [21] as synthons. In contrast, in the divergent approach two or more distinct strands are prepared with synthons with orthogonal protecting groups or from commercial sources [20, 22]. In addition, 2′-O-silylribonucleosides have been used to synthesize asymmetric double oligonucleotide strands [23].

Synthetic branched oligonucleotides have been applied for several purposes. Initially, most of the interest in this area was focused on the study of branched oligoribonucleotides as splicing intermediates of eukaryotic mRNAs [24–26]. Moreover, branched oligonucleotides show high affinity for single-strand oligonucleotides to form alternated strand triplexes [27–29]. Recently, branched oligonucleotides have been used as building blocks in the synthesis of new nanostructures [30–34]. Multilabelled oligonucleotides containing branching points have been described to increase the sensitivity of hybridization experiments [35]. 

Here, we synthesized branched RNA structures (Figure 1(b)) and evaluated their capacity to inhibit the tumour necrosis factor (TNF-*α*) protein, which is involved in the apoptosis, inflammation, and immunity processes [36]. We reasoned that branched siRNA could provide a new RNA architecture for RNA interference activity. Given that the use of symmetric branching units is compatible with most of the modifications described to enhance the inhibitory capacity of siRNA, the molecules described here may provide a starting point for further modifications.

## 2. Experimental Section

### 2.1. Oligonucleotides

The following RNA sequences were obtained from commercial sources (*Sigma-Proligo*, *Dharmacon*): sense or passenger scrambled 5′-CAGUCGCGUUUGCGACUGG-dT-dT-3′, antisense or guide scrambled 5′-CCAGUCGCAAACGCGACUG-dT-dT-3′, antisense or guide anti-TNF-*α*: 5′-GAGGCUGAGACAUAGGCAC-dT-dT-3′, and sense or passenger anti-TNF-*α*: 5′-GUGCCUAUGUCUCAGCCUC-dT-dT-3′. RNA monomers in capital letters, dT represents thymidine. The anti-TNF*α* siRNA was previously described to efficiently downregulate murine TNF*α* mRNA [36].

**Figure 2:**
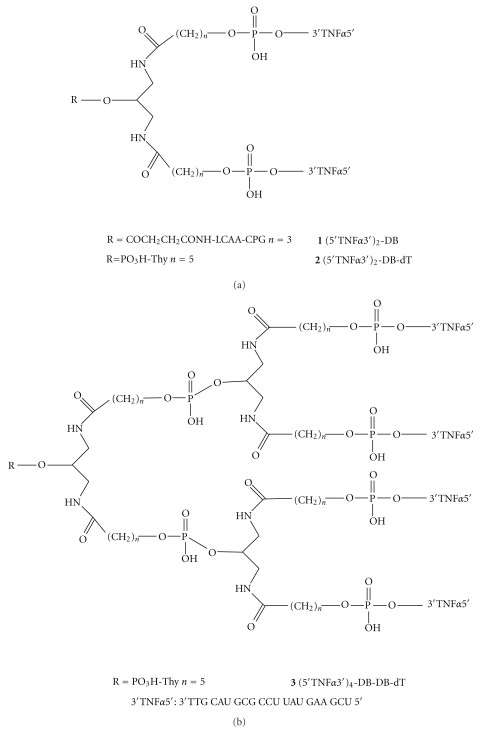
Schematic representation of the chemical structure of the branching units of the oligonucleotides described in this study.

Figure 2 refers to the branched RNA structures synthesized in this study. DB stands for the symmetric doubler phosphoramidite obtained from commercial sources (*Glen Research*). Guanosine was protected with the dimethylaminomethylidene group, cytidine with the acetyl group, and adenosine with the benzoyl group. *t*-Butyldimethylsilyl (TBDMS) group was used for the protection of the 2′-OH function of the RNA monomers. The phosphoramidites were dissolved in dry acetonitrile (0.1 M), and a modified cycle was used with increased coupling time to 10 min. Oligoribonucleotide **1** was synthesized on a CPG solid support with a symmetric branching unit of two arms containing two DMT-protected hydroxyl groups, as described in [31]. Oligoribonucleotides **2** and **3** were synthesized using standard low-volume polystyrene thymidine columns. After the solid-phase synthesis, the supports were treated with concentrated aqueous ammonia-ethanol (3 : 1) for 1 h at 55°C. After filtration of the supports, the solutions were evaporated to dryness. The residue was dissolved in 85 *μ*L of 1 M tetrabutylammonium fluoride (TBAF) in tetrahydrofuran (THF) for 12 h. Then, 85 *μ*L of 1 M of triethylammonium acetate was added and the oligoribonucleotides were desalted on a NAP-10 column using water as eluent. The compounds were purified by HPLC under the following conditions. Column: Nucleosil 120–10 C_18_ (250  ×  4 mm); 20-min linear gradient from 15% to 100% B (DMT ON conditions); flow rate 3 mL/min; solution A was 5% acetonitrile in 0.1 M aqueous triethylammonium acetate (TEAA) buffer and B 70% acetonitrile in 0.1 M aqueous TEAA. The purified products were analyzed by MALDI-TOF mass spectrometry. Yields (0.2 *μ*mol scale synthesis) were between 5–10 OD units at 260 nm.

### 2.2. Thermal Denaturation Studies

The thermal melting curves for duplexes of the oligoribonucleotides **1**–**3** and their unmodified RNA complementary strands (guide strand) were performed following the absorption change at 260 nm. Samples were heated from 20°C to 80°C, with a linear temperature ramp of 0.5°/min in a JASCO V-650 spectrophotometer equipped with a Peltier temperature control. Sample concentration of the samples was around 2 *μ*M. All the measurements were repeated three times and conducted in 15 mM HEPES 1 mM Mg(OAc)_2 _ and 50 mM KOAc pH 7.4.

### 2.3. Cell Culture, Transfection, and Cellular Assays

HeLa cells were cultured under standard conditions (37°C, 5% CO_2_, Dulbecco's Modified Eagle Medium, 10% fetal bovine serum, 2 mM L-glutamine, supplemented with penicillin (100 U/mL) and streptomycin (100 mg/mL)). All *in vitro* experiments were performed at 40–60% confluence. HeLa cells were transfected with 250 ng of a plasmid expressing murine TNF-*α* using lipofectin (*Invitrogen*), following the manufacturer's instructions. One hour after transfection cells were transfected with 100 nM double strand concentration of siRNA against TNF-*α*, using oligofectamine (*Invitrogen*). Previously, siRNA duplex annealing was performed by mixing modified (**1**, **2**, **3**) and unmodified passenger strands (**unm**) with the appropriate amount of the corresponding unmodified guide strand. 

TNF-*α* concentration was determined from cell culture supernatant by enzyme-linked immunosorbent assay kit (*Bender MedSystems*) following the manufacturer's instructions. The inhibitory capacity of the siRNA duplexes is expressed as double strand concentration for comparative purposes. A 100 mM double strand concentration is equivalent to a 50 nM concentration of two-branched siRNA (**1** or **2**) and to 25 nM of four-branched siRNA (**3**).

## 3. Results and Discussion

### 3.1. Oligonucleotide Synthesis

**Figure 3:**
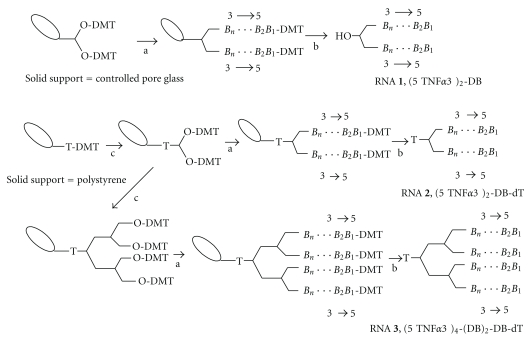
Outline of the synthesis of branched RNA oligonucleotides **1**,** 2**, and **3** with two and four arms with identical sequence. (a) Assembly of the RNA sequence by standard solid-phase methods; (b) removal of protecting groups and release of the RNA molecule from solid support; (c) addition of the symmetrical branching phosphoramidite.

**Figure 4:**
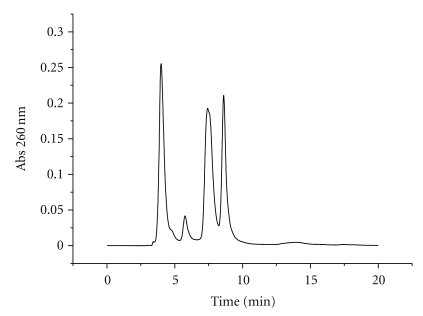
HPLC profile of DMT-containing oligonucleotide **2** with two arms. Truncated sequences without DMT groups had a retention of less than 5 min. Fraction eluting between 7-8 min contained oligonucleotides with a single DMT group. The last fraction contained the desired sequence with two DMT groups.

**Figure 5:**
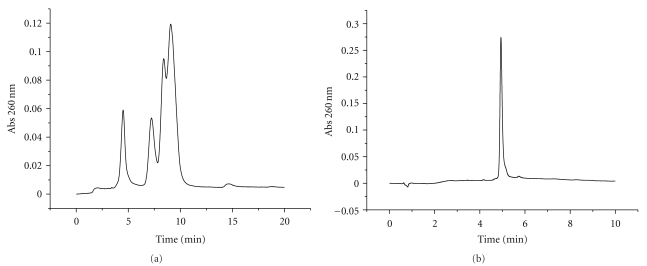
HPLC purification of oligonucleotide **3** with four arms. (a) HPLC profile of DMT-containing oligonucleotide **3**. The last fraction contained the desired sequence with four DMT groups; (b) analytical HPLC of purified oligonucleotide **3 **after removal of DMT groups.

In order to prepare branched RNA for RNA interference, the potential steric hindrance of the branching unit with RISC must be considered. As the passenger strand is removed from the siRNA duplex upon binding to RISC, we introduced the branching modification at the protruding 3′-end of the sense strand. This position has been demonstrated to allow the introduction of a large number of modifications without affecting the inhibitory capacity of siRNA [6, 7]. We thus designed branched oligonucleotide sequences **1**–**3** of the passenger strand of a siRNA directed against TNF-*α* (Figure 2). Sequence **1 **was synthesized using a controlled pore glass (CPG) solid support containing a symmetric doubler [31], as shown in Figure 3. Sequences **2** and **3 **with two or four strands, respectively, were synthesized on a low-volume polystyrene support (LV200) functionalized with dimethoxytrityl- (DMT-) thymidine. The commercially available symmetric doubler phosphoramidite was used to introduce two and four branches on the 3′-position of the starting thymidine (Figure 3). Sequences were assembled using standard protocols for RNA synthesis. The 2′-OH function of ribonucleosides was protected with the *t*-butyldimethylsilyl (TBDMS) group. Coupling yields, determined by the absorbance of the DMT cation released in each synthesis step, were more efficient (98%) on low-volume (LV200) polystyrene supports than on CPG support (95%). After assembly of the sequences, the DMT-containing oligonucleotides were released from the supports with ammonia, and the resulting compounds were treated with fluoride to remove the TBDMS groups. HPLC analysis of the resulting products is shown in Figures 4 and 5. Several peaks were observed for the synthesis of the two-branch RNA sequences (**1** and **2**; Figure 4). Truncated sequences without DMT groups eluted between 3–5 min. A fraction containing oligonucleotides with a single DMT group was eluted next, and the last fraction contained the desired sequence with two DMT groups. Mass spectrometry analysis (Table 1) and electrophoresis analysis confirmed the mass and size of the desired branched oligoribonucleotides.

Figure 5 shows the HPLC profile of the mixture obtained in the synthesis of the four-branch RNA sequence (**3**). In this case, three peaks in the DMT-containing area were observed. Although resolution of these peaks was not as good as in the previous case, the last eluting peak corresponded to the desired tetra-DMT compound (**3**). The purified compound had the expected molecular weight, was homogeneous by analytical HPLC (Figure 5), and showed the correct migration in polyacrylamide gel electrophoresis (PAGE). 

### 3.2. Thermal Denaturation Studies

The melting temperatures of the branched siRNA duplexes formed by annealing of equimolar amounts of sequences **1**–**3** with unmodified passenger strand are shown in Table 1. Duplex **1 **had the lowest melting temperature, which was 3.5°C lower than the unmodified duplex (Table 1). Duplex **2** melted 1.5°C lower than the unmodified duplex. In contrast, duplex **3** had similar melting temperatures as the unmodified duplex. The small decrease in melting temperatures of the two-branched siRNA structures is possibly due to a steric effect in the branching point that holds the two duplex strands in close proximity. The four-stranded architecture had a larger separation between strands as a result of the introduction of 3 branching units, thus the resulting duplexes showed greater similarity to the unmodified duplex. Thus we believe that the small destabilizing effect observed in the two-branched RNA duplexes could be optimized in further experiments by adding a linker between the branching unit and the RNA strands, as described by Grimau et al. [31].

### 3.3. Cell Culture, Transfection, and Cellular Assays

**Figure 6:**
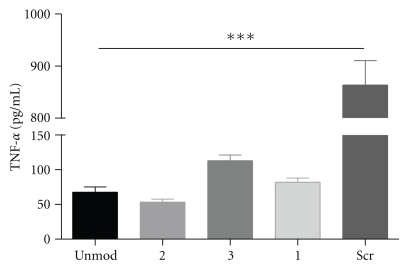
Inhibitory capacity of branched anti-TNF-*α* siRNAs. The inhibitory capacity of the siRNA duplexes are expressed as double strand concentration for comparative purposes. A 100-nM double strand concentration is equivalent to a concentration of 100 nM of unmodified siRNA duplex, 50 nM of siRNA **1**, and **2** and 25 nM of siRNA **3**. Transfection of siRNAs was carried out using oligofectamine. Values are represented as the average ±ES, *n* = 3 and are compared to a scrambled sequence. ***P < .001, ANOVA Test, Bonferroni post-test.

Tumor necrosis factor (TNF-*α*) was selected as a target for RNA interference studies. This protein is a major mediator of apoptosis as well as inflammation and immunity, and it has been implicated in the pathogenesis of a wide spectrum of human diseases. Consequently, inhibition of this protein is of particular relevance. Modified oligoribonucleotides (**1**–**3**) were annealed with equimolar amounts of the unmodified guide, and the resulting duplexes were used to inhibit the expression of TNF-*α* gene. HeLa cells were transfected first with the murine TNF-*α* plasmid using lipofectin, and 1 h later they were cotransfected with the siRNA duplex using oligofectamine. After 24 h, cellular TNF-*α* production was analyzed by enzyme-linked immunosorbent assay (ELISA). The inhibitory capacity of the siRNA duplexes is shown in Figure 6. To compare the efficiency of each siRNA to inhibit TNF-*α*, we normalized the data taking in account the number of strands of each siRNA. Thus, a 100 nM double strand concentration is equivalent to 100 nM of unmodified siRNA duplex, 50 nM of siRNA **1**, and **2**, and 25 nM of siRNA **3**. Figure 6 shows that the inhibitory capacity of the branched structures was maintained similar to that of the unmodified duplex. This result indicates that the branched siRNAs described here are compatible with RNA interference machinery, and thus the RISC complex binds to branched RNA structures in a similar way as to the linear RNA duplexes shown in Figure 1. Two-stranded RNA duplexes (**1** and **2**) were more efficient than four-stranded ones (**3**). In addition, siRNA **1** showed slightly greater efficiency at inhibiting TNF-*α* than siRNA duplex **2**. This small difference may be related to the lower melting temperature of the former (Table 1).

## 4. Conclusions

For several years, research has focused on chemical modifications and delivery technologies to improve the pharmacokinetic properties of siRNA. Many of the chemically modified siRNA with interesting inhibitory capacity contain one or multiple modifications in the sugar, nucleobases, and phosphate linkages or at the 3′- or 5′-ends. In addition to these modifications, duplex architecture of siRNA itself is also relevant, and several modifications have been reported to show satisfactory inhibitory capacity. Here we demonstrate that branched siRNA is compatible with RNAi and that, when transfected with cationic lipids, siRNA has similar inhibitory capacity than unmodified duplex siRNA. Although the potency of branched siRNA containing two or four strands was not increased, we consider it a suitable starting point for further development. Given that the method described here is compatible with most of the RNA modifications described to date, these compounds may be further functionalized to obtain more potent siRNA derivatives. In addition, they offer an internal mid position that could be suitable for attachment to delivery systems. In this regard, optimization of the branching approach for the synthesis of asymmetric branched siRNAs may lead to the development of siRNA for the combined inhibition of multiple targets. These asymmetric siRNA duplexes carrying two RNA sequences attached or bound to an appropriate delivery system will insure the 1 : 1 ratio of two RNA sequences for the combined inhibition of two genes that may improve the treatment of a particular disease.

## Figures and Tables

**Table 1:** Mass spectrometry data on modified passenger strand and melting temperatures of siRNA duplexes formed by oligonucleotides **1**, **2**, and **3** and the linear unmodified control sequences. Buffer conditions: 15 mM HEPES, 1 mM magnesium acetate, 50 mM potassium acetate pH 7.4.

N°	oligonucleotides	MS (expected)	MS (found)	Tm (°C)	Δ*T* (°C)
**1**	(5′TNF*α* 3′)_2_-DB	13508	13504	79.8	−3.5
**2**	(5′TNF*α* 3′)_2_-DB-dT	13842	13847	82.5	−1.7
**3**	(5′TNF*α* 3′)_4_-(DB)_2_-DB-dT	27798	27823	84.3	+0.1
**Unmodified**	Passenger TNF*α*	n.d.	n.d.	84.2	0
